# DAILY SUBJECTIVE NEARNESS TO DEATH AND AFFECTIVE DYNAMICS

**DOI:** 10.1093/geroni/igad104.0981

**Published:** 2023-12-21

**Authors:** Shevaun Neupert, Lyndsey Graham

**Affiliations:** North Carolina State University, Raleigh, North Carolina, United States; North Carolina State University, Raleigh, North Carolina, United States

## Abstract

A core tenet of Socioemotional Selectivity Theory is that perception of time remaining in life plays a key role in the selection and pursuit of emotion regulation goals. The current study examined this idea within a daily diary framework, where subjective perceptions of nearness to death were reported on consecutive days. Age differences in the within-person coupling between daily subjective reports of nearness to death and daily affective dynamics were tested. A 14-day daily diary study of 440 adults (M age = 65, range 50-85) in the U.S. was conducted where participants reported on their subjective nearness to death, positive affect, and negative affect each day. Multilevel models were conducted separately for positive and negative affect. Being older and feeling farther away from death on average were each associated with higher levels of positive affect. Daily negative affect was predicted by both within-person and between-person constructs. On days when people reported feeling subjectively closer to death than their own average, they also experienced an increase in negative affect. A significant interaction revealed that this effect was particularly strong among older adults (aged 65+). These results suggest that daily manifestations of perceptions of limited time remaining in life are salient for daily affective dynamics, and that this salience is particularly pronounced among older adults relative to those in midlife.

